# Areca nut chewing and metabolic syndrome: evidence of a harmful relationship

**DOI:** 10.1186/1475-2891-12-67

**Published:** 2013-05-20

**Authors:** Kashif Shafique, Mubashir Zafar, Zeeshan Ahmed, Naveed Ali Khan, Muhammad Akbar Mughal, Fauzia Imtiaz

**Affiliations:** 1 Department of Community Medicine, Dow University of Health Sciences, Karachi, Pakistan; 2 Institute of Health & Wellbeing, Public Health, University of Glasgow, 1 Lilybank Gardens, Glasgow, G12 8RZ, UK; 3 Department of Surgery, Dow University of Health Sciences, Karachi, Pakistan; 4 Department of Physiology, Karachi Medical and Dental College, Karachi, Pakistan; 5 Department of Biochemistry, Dow International Medical College, Karachi, Pakistan

**Keywords:** Areca nut chewing, Metabolic syndrome, Obesity, Lipid abnormalities

## Abstract

**Background:**

There is some evidence which suggests that areca nut chewing has a relationship with metabolic syndrome. Areca nut chewing is continue to increase and so is the metabolic syndrome which is a major cause of cardiovascular mortality in developing countries. The aim of this study was to determine the relationship of raw areca nut and areca nut chewing with tobacco additives and metabolic syndrome.

**Methods:**

This cross sectional study was conducted on population of Karachi, Pakistan. Simple random sampling was implied using the voter list as a sampling frame. A detailed questionnaire about the demographic details of all subjects was filled and an informed consent obtained for blood sampling. Logistic regression analyses were carried out to investigate the relationship between areca nut chewing and metabolic syndrome.

**Results:**

Of the 1070 individuals, 192(17.9%) had metabolic syndrome with significantly higher (p-value <0.001) prevalence among females (26.3%) compared with males (11.4%). Eight individuals (11.1%) among non users had metabolic syndrome while significantly higher (p-value <0.001) proportion of both, raw areca nut users (n = 67, 29%) and areca users with tobacco additives (n = 45, 38.5%) had metabolic syndrome.

The crude odds ratio for central obesity among raw areca nut users was 1.46 (95% CI 1.07-1.98) and among areca nut users with tobacco additives was 2.02 (95% CI 1.36-3.00), hypertension among raw areca nut users group was 1.31(0.96-1.78) and among areca nut users with tobacco additives group was 2.05 (95% CI 1.38-3.04). A significant positive association of raw areca nut chewing and metabolic syndrome was found among males (crude OR 2.74, 95% CI 1.52-4.95) and females (crude OR 3.80, 95% CI 2.32-6.20). Similarly, a significant positive association was found with regard to raw areca nut with tobacco additives chewing among males (crude OR 5.46, 95% CI 2.73-10.91) and females (crude OR 4.32, 95% CI 2.41-7.72). These associations remained significant adjustment for age, social class.

**Conclusions:**

This study suggests a harmful relationship between areca nut chewing and metabolic syndrome. The deleterious effects were even stronger among areca nut chewer with tobacco additives. Further research with longitudinal data might help to understand the temporal relationship between areca nut chewing and metabolic syndrome.

## Background

The metabolic syndrome has become one of the major public-health challenges worldwide [1]. The metabolic syndrome is defined as a cluster of dangerous risk factors (particularly for heart disease), which include diabetes, pre-diabetes (raised blood glucose level), abdominal obesity, high cholesterol level and high blood pressure [1]. There has been growing interest in this group of closely related cardiovascular risk factors because this group is at significantly higher risk of developing both cardiovascular and cerebrovascular diseases. Areca nut chewing has been recently linked with metabolic syndrome [2]. Approximately 600 million individuals chew Areca nut worldwide making it fourth commonest substance after nicotine, ethanol and caffeine [3]. Areca nut chewing has already been linked with the development of oral and oesophageal cancer, hepatocellular carcinoma [4-6] and more recently with metabolic syndrome [7-9].

Two earlier studies have suggested that areca nut chewers are significantly more likely to have metabolic syndrome with up to two-fold increase in risk compared with non-users [10,11]. Furthermore, some other studies have attempted to examine the relationship between areca nut chewing and components of metabolic syndrome particularly obesity [12,13] and diabetes mellitus [14,15]. Three studies reported increased risk of general and central obesity among areca nut chewers compared with non-users [11-13]. The increased risk reported in these studies ranged from 30% to two-fold [11-13]. Additionally, two other studies reported 30% increase odds of diabetes mellitus and hyperglycaemia among areca nut users compared with non-users [14,15].

It is important to understand whether the relationship between areca nut and metabolic syndrome remains similar between different types of areca nuts [3,16]. Recently, there is a trend of areca nut chewing with additives particularly nicotine - which may be worse than the raw areca nuts. Therefore, it is important to examine the effects of areca nut chewing (with and without additives) on metabolic profiles of health individuals. There is no previous evidence examining such effects of areca nut chewing with tobacco additives and metabolic syndrome. Therefore, the objective of this study is to determine the relationship of areca nut chewing with or without additives and metabolic syndrome.

## Methods

This was a cross sectional study conducted in new Karachi town, Karachi, Pakistan an area of approximate population of 1 million. Individuals were selected through simple random sampling from the sampling frame of voter list. Sample was calculated using the WHO software for sample size determination in health studies. Sample size was calculated based on proportion of prevalence of areca nut chewing as reported by a previous study [3]. To calculate sample size by using proportion of 28.9% at confidence level of 95% and bound of error 3%, the estimated sample size required was 1475. Total 1500 individuals were recruited for participation in the study. Participants were included between the ages of 16 to 75 years of age. Pre-tested self administered questionnaire which included socio-demographic details, lifestyle habits and history of any known chronic disease, current and past use of medications. A trained data collector was hired, he collected the data and serum blood samples were drawn and sent to laboratory for carrying out blood biochemical and haematological investigations.

International Diabetic Federation (IDF) values were used for central obesity, raised triglyceride, reduced HDL, raised BP and Hyperglycaemia [17]. According to the new definition for a person to be defined as having the metabolic syndrome must have central obesity plus any two of the four factors which include raised triglyceride (TG) (≥1.7 mmol/L or specific treatment for this lipid abnormality), reduced High Density Lipoprotein (HDL) cholesterol (<1.03 mmol/L in males and <1.29 mmol/L in females or specific treatment for this lipid abnormality), raised blood pressure (systolic blood pressure ≥130 or diastolic blood pressure ≥85 or treatment of previously diagnosed hypertension) and raised fasting plasma glucose (≥5.6 mmol/L or previously diagnosed type 2 diabetes) [17]. To determine the abdominal obesity we used South Asian specific cut off in which a waist circumference for male ≥ 90 cm and female ≥80 was considered as obese [17]. Ethical approval was given by an independent ethics committee at Afra General Hospital, Faisalabad, Pakistan. Consent form and questionnaire was developed in English and Urdu to disseminate the objectives of this research study to the participants and written consent was obtained prior to interview.

### Investigations

Blood samples were taken to investigate complete blood count, lipid profile and blood glucose. Used Sysmex Pouch counter (An automated machine by S Ejaz uddin & co) for complete blood count. For complete blood count, we took 5 ml of blood in a purple top vaccutainer (containing ethylene diamine tetra acetic acid in it in it) and after 5 min mixing on rotator, the sample was ran in machine and results were obtained.

### Statistical analysis

Stata software version 11 (StataCorp, College Station, TX, USA) were used to analyse the collected data. Participants were divided into three categories based on their areca nut chewing habits, i.e. non users, raw areca nut user and areca nut with additives used. Central obesity was diagnosed by using the waist circumference measurements and ethnicity specific values cut offs [17]. Mean and standard deviation were calculated for continuous variables and frequency for categorical variables. Univariate and multivariate logistic regression were used to estimate crude and adjusted odds ratio for metabolic syndrome using demographic and areca nut chewing co-variates. We also compared the gender differences in existence of metabolic syndrome and its components. Age-stratified analysis was also carried out to examine the relationship between areca nut chewing and metabolic syndrome. All the significance tests were two tailed with a significance level 0.05%.

## Results

### Basic characteristics of study sample

In total, 1500 individuals were invited for this study, 1089 accepted the invitation (response rate 72.6%). Nineteen individuals were not eligible due to current febrile illness while areca nut chewing, haematological and biochemistry data were missing for 10 individuals so the final analysis included the data of 1070 apparently healthy individuals. Of the 1070, 348 (32.5%) were areca nut users. Among these 348, 117 (33.6%) were users of areca nut with tobacco additives. Males were slightly more likely to be non-users and female were slightly more likely to be areca nut users with tobacco additives (p-value 0.05). Generally, there were no significant differences in age between three groups (p-value 0.95) and total cholesterol level (p-value 0.31). Areca nut users with tobacco additives had significantly, higher waist circumference (p-value <0.001), higher systolic (p-value <0.001) and diastolic blood pressure (p-value 0.003), higher blood glucose level (p-value <0.001), higher serum triglyceride level (p-value 0.004) and lower high density lipoprotein (p-value 0.03). Basic characteristics of study sample have been summarized in Table 1.

**Table 1:** Baseline characteristics of study sample based on areca nut chewing categories

**Characteristics**	**Non-users**	**Areca nut chewers**		P-value*
		**Raw areca nut users**	**Areca with tobacco additives**	
**Total participants, n(%)**	722(67.5)	231(21.6)	117(10.9)	_
**Male sex, n(%)**	419(70.0)	126(21.0)	54(9.0)	0.05
**Age, mean (SD)**	33.8(15.3)	33.9(15.6)	33.4(14.7)	0.95
**Age, n(%)**				
Age 16-39	470(67.3)	151(21.6)	77(11.0)	0.95
Age 40-49	99(66.9)	32(21.6)	17(11.5)	
Age 50-59	87(69.1)	24(19.1)	15(11.9)	
Age 60-75	66(67.4)	24(24.5)	8(8.2)	
**Waist circumference, mean(SD)**	80.3(12.1)	82.1(12.4)	84.0(12.3)	0.003
**Central obesity, n(%)**	231(32.0)	94(40.7)	57(48.7)	<0.001
**Systolic blood pressure, mm Hg, mean(SD)**	123.6(19.0)	126.5(20.4)	131.0(21.4)	<0.001
**Diastolic blood pressure, mm Hg, mean(SD)**	71.6(9.5)	73.1(9.8)	74.5(9.6)	0.003
**Cholesterol level, mmol/L, mean(SD)**	5.6(1.1)	5.8(1.2)	5.7(1.0)	0.31
**High density lipoprotein, mmol/L, mean(SD)**	1.4(0.4)	1.3(0.4)	1.3(0.4)	0.03
**Serum triglyceride level, mmol/L, mean(SD)**	1.4(0.9)	1.6(1.1)	1.6(1.0)	0.004
**Blood glucose level, mmol/L, mean(SD)**	4.8(0.9)	5.0(1.2)	5.3(2.0)	<0.001
**Social class**				
Professionals	158(67.5)	44(18.8)	32(13.7)	<0.001
Non-manual workers	115(42.3)	113(41.5)	44(16.2)	
Manual workers	449(79.6)	74(13.1)	41(7.3)	

*p-value for categorical variables was calculated by chi squared test and with ANOVA for continuous variables.

### Areca nut chewing and metabolic syndrome

Of the 1070 individuals, 192 (17.9%) had metabolic syndrome with significantly higher (p-value <0.001) prevalence among females (26.3%) compared with males (11.4%). Eight individuals (11.1%) among non users had metabolic syndrome while significantly higher (p-value <0.001) proportion of both, raw areca nut users (n = 67, 29%) and areca users with tobacco additives (n = 45, 38.5%) had metabolic syndrome. Age-adjusted prevalence of metabolic syndrome significantly differed between male and female areca nut users. A significant positive association was found with regard to raw areca nut chewing among males (crude OR 2.74, 95% CI 1.52-4.95) and females (crude OR 3.80, 95% CI 2.32-6.20). Similarly, a significant positive association was found with regard to raw areca nut with tobacco additives chewing among males (crude OR 5.46, 95% CI 2.73-10.91) and females (crude OR 4.32, 95% CI 2.41-7.72). These associations remained significant adjustment for age, social class (Table 2).

**Table 2:** The relationship between areca nut chewing and metabolic syndrome by gender

	**Metabolic syndrome**				**Metabolic syndrome**			
	**Males**				**Females**			
	**Univariate analysis**	**P-value**	Multivariate analysis *	**P-value**	**Univariate analysis**	**P-value**	Multivariate analysis *	**P-value**
	**OR (95% CI)**		**OR (95% CI)**		**OR (95% CI)**		**OR (95% CI)**	
**Areca nut chewing**								
Non users	Reference		Reference		Reference		Reference	
Raw areca nut chewers	2.74 (1.52-4.95)	0.001	2.85 (1.47-5.53)	0.002	3.80 (2.32-6.20)	<0.001	4.22 (2.40-7.41)	<0.001
Areca nut with tobacco additives	5.46 (2.73-10.91)	<0.001	5.41 (2.49-11.74)	<0.001	4.32 (2.41-7.72)	<0.001	4.37 (2.31-8.26)	<0.001

*Multivariate models included age and social class.

We also conducted age stratified analysis to examine the relationship between areca nut chewing and metabolic syndrome. Among those age <40 years, areca nut chewers were 4.3 time more likely to have metabolic syndrome (OR 4.30, 95% CI 2.74-6.74) after adjusting for social class, compared to non-chewers. Likewise, those age ≥40 years, areca nut chewers were four times more likely to have metabolic syndrome (OR 4.03, 95% CI 2.74-6.74), after accounting for social class, compared to non-chewers.

### Areca nut chewing and components of metabolic syndrome

The crude odds ratio for central obesity among raw areca nut users was 1.46 (95% CI 1.07-1.98) and among areca nut users with tobacco additives was 2.02 (95% CI 1.36-3.00 p-value for trend <0.001). This association slightly attenuated but remained significant (p-value for trend 0.02) after adjustment for age, gender and social class. The crude odds ratio for hypertension among raw areca nut users group was 1.31 (0.96-1.78) and among areca nut users with tobacco additives group was 2.05(95% CI 1.38-3.04, p-value for trend <0.001) while the odds ratio after controlling for possible confounding was 1.23 (95% CI 0.89-1.70) and 1.87 (95% CI 1.25-2.81, p-value 0.003), respectively. Similarly, raw areca nut users had significantly, reduced HDL (OR 1.72, 95% CI 1.72-2.33), hypertriglyceridemia (OR 1.73, 95% CI 1.24-2.41) and hyperglycemia (OR 1.65, 95% CI 1.11-2.46). These associations remained significant after controlling for age, gender and social class (Table 3). The pattern of associations of metabolic syndrome remains similar among areca nut users with tobacco additives and even higher odds ratio compared to raw areca nut chewers (Table 3).

**Table 3:** Relationship between areca nut chewing and components of metabolic syndrome

			**Areca nut chewers**	
	**Non chewers**	**Raw areca chewers**	**Areca with tobacco additives**	***P-value for trend***
**Total participants**	722	231	117	
**Central Obesity**				
Obese, n(%)	231 (32.0)	94 (40.7)	57 (48.7)	
Odds Ratio (95% CI)^a^	reference	1.46 (1.07-1.98)	2.02 (1.36-3.00)	<0.001
Odds Ratio (95% CI)^b^	reference	1.42 (0.97-2.06)	1.65 (1.03-2.63)	0.015
**Raised blood pressure**				
Raised BP cases, n(%)	234 (32.4)	89 (38.5)	58 (49.6)	
Odds Ratio (95% CI)^a^	reference	1.31 (0.96-1.78)	2.05 (1.38-3.04)	<0.001
Odds Ratio (95% CI)^b^	reference	1.23 (0.89-1.70)	1.87 (1.25-2.81)	0.003
**Reduced HDL**				
Reduced HDL cases, n(%)	233 (32.8)	104 (45.0)	58 (50.0)	
Odds Ratio (95% CI)^a^	reference	1.72 (1.27-2.33)	2.06 (1.39-3.06)	<0.001
Odds Ratio (95% CI)^b^	reference	1.55 (1.11-2.17)	1.66 (1.09-2.54)	0.003
**Hypertrigylceridemia**				
Hypertriglyceridemia cases, n(%)	150 (20.8)	72 (31.2)	41 (35.0)	
Odds Ratio (95% CI)^a^	reference	1.73 (1.24-2.41)	2.06 (1.35-3.13)	<0.001
Odds Ratio (95% CI)^b^	reference	1.82 (1.28-2.59)	2.04 (1.32-3.16)	<0.001
**Hyperglycemia**				
Hyperglycemia cases, n(%)	88 (12.2)	43 (18.6)	26 (22.2)	
Odds Ratio (95% CI)^a^	reference	1.65 (1.11-2.46)	2.06 (1.26-3.36)	0.001
Odds Ratio (95% CI)^b^	reference	1.48 (0.97-2.27)	1.80 (1.08-3.00)	0.011

a = unadjusted odds ratio, b = estimates adjusted for age, gender and social class.

## Discussion

The present study suggests that areca nut chewing has significant relationship with metabolic syndrome. It was notable that areca nut chewers with tobacco additives had higher odds of metabolic syndrome compared with raw areca users. Furthermore, areca nut chewing was associated with increased odds of central obesity, hypertension, hyperglycemia and dyslipidemia. These relationships remain unchanged even after adjusting for age, gender and social class. This study also highlighted that areca nut chewing may have differential effects on metabolic syndrome and its component with more deleterious effects among females.

The findings of this study are consistent in terms of direction of association that areca nut chewers are more likely to have metabolic syndrome, however the effect sizes – both for metabolic syndrome and components of metabolic syndrome - reported in this study are generally greater than the earlier studies [10,11]. The odds of having obesity and diabetes mellitus among areca nut users are also significantly higher in our study compared with earlier studies, where some examined obesity [11-13] and others examined diabetes mellitus [14,15]. Although the underlying mechanism of these associations remains unclear, however, several possible pathways have been suggested earlier. The suppression of hunger reported by some long term areca nut chewers may induce a state of malnutrition which could lead towards vitamin-D deficiency [18]. Vitamin-D has been identified as risk factor for many systemic disease including MetS and diabetes mellitus [19]. In an experimental study, young adult CD1 mice were fed areca nut in standard feed for 2–6 days. On comparison with controls, these mice had significantly higher incidence of diabetes mellitus at the age of six months [20,21]. A further consideration is the relationship between areca nut chewing and obesity which has been reported by many population-based studies [22]. Arecoline (an alkaloid which is a major component of areca nuts) by interfering with gamma-aminobutyric acid receptors, can suppress the appetite while increasing postprandial carbohydrate use [23].

Interestingly, the effect sizes for metabolic syndrome and its components observed in this study for raw areca nut chewers are fairly consistent as reported earlier. However, areca nut chewers with tobacco additives were significantly more likely to have metabolic syndrome. There is no earlier study which reported on type of areca and its relationship with metabolic syndrome so we are unable to compare these estimates with any known figures. However, it is reasonable to assume that tobacco content of these nuts may be mediating some additional metabolic changes leading to metabolic syndrome.

The lifestyle factors, for instance, dietary habits and physical activity, may also be contributing to the development of metabolic syndrome. However, we further adjusted for social class of individuals which covers some of these aspects of lifestyle but the apparent relationship between areca nut chewing and metabolic syndrome remain unchanged. Simultaneously, the role of confounding factors such as lower education and health risk behaviours, which increase the risk of metabolic syndrome may also have some effect on this relationship but as social class was derived by occupation, any such effect may have been accounted by adjusting for social class. However, we can not completely rule out the possibility of some residual confounding by lower education and health risk behaviour on this relationship between areca nut chewing and metabolic syndrome.

### Strength and limitations

This is the first study which examined the role of different types of areca nuts on metabolic syndrome and its components. Most previous studies only included male subjects while this study also had a fairly large sample of females which provides further insight to the differential distribution of metabolic syndrome and its components between males and females. There are few limitations of this study which are worth to be reported. We aimed to obtain a sample of 1475 individuals; however, the required sample size was not achieved. Although, this was a limitation of this study, but the association between areca nut chewing and metabolic syndrome remains statistically significant even with this smaller sample. So that suggests a convincing association between areca nut chewing and metabolic syndrome may be present, and a larger study may provide more precise estimate for this relationship. This study used cross sectional design, which is not the best approach to examine any causal relationships, however this study does raise some questions about the different types of areca nuts and its relationship which needs further investigation. Future research with prospective design may provide robust evidence on role of areca nut chewing in development of metabolic syndrome. Finally some element of bias can not be excluded as individuals with higher consumption of areca nut may have other systemic symptoms or repeated infections which may lead to higher utility of health care services. However, random invitations might have eliminated some of the effects caused by chance.

## Conclusion

This study suggests a harmful relationship between areca nut chewing and metabolic syndrome. The deleterious effects were even stronger among areca nut chewers with tobacco additives. Further research with longitudinal data might help to understand the temporal relationship between areca nut chewing and metabolic syndrome.

## Competing interests

The authors declare that they have no competing interests.

## Authors’ contributions

All authors designed the study; FZ collected the data, KS carried out statistical analyses; all authors contributed to interpreting the results; KS, MZ, ZA, MAM, NAK and FZ drafted the manuscript; all authors saw and approved the final manuscript.
